# Dynamic Metabolic Control: From the Perspective of Regulation Logic

**DOI:** 10.35534/sbe.2023.10012

**Published:** 2023-08-28

**Authors:** Tian Jiang, Chenyi Li, Yuxi Teng, Jianli Zhang, Diana Alexis Logan, Yajun Yan

**Affiliations:** School of Chemical, Materials, and Biomedical Engineering, College of Engineering, The University of Georgia, Athens, GA 30602, USA

**Keywords:** Dynamic regulation, Control logic, Feedback control, Oscillation

## Abstract

Establishing microbial cell factories has become a sustainable and increasingly promising approach for the synthesis of valuable chemicals. However, introducing heterologous pathways into these cell factories can disrupt the endogenous cellular metabolism, leading to suboptimal production performance. To address this challenge, dynamic pathway regulation has been developed and proven effective in improving microbial biosynthesis. In this review, we summarized typical dynamic regulation strategies based on their control logic. The applicable scenarios for each control logic were highlighted and perspectives for future research direction in this area were discussed.

## 1. Introduction

Metabolic engineering manipulates microbial metabolism to produce value-added products and maximize productivity to fulfill the high demands of industrial production [1,2]. Recent years have witnessed the development of metabolic engineering for the biosynthesis of natural products [3], pharmaceuticals [4], cosmetics [5], and bulk chemicals [6] in microbial cell factories. By introducing heterologous pathways into the microbial hosts, such as *Escherichia coli, Bacillus subtilis, Saccharomyces cerevisiae*, and *Corynebacterium glutamicum* [6–10], inexpensive feedstocks can be converted into valuable products. For example, Zhang et al. constructed a biosynthetic pathway for the production of the anti-cancer drug vinblastine in yeast using glucose as the carbon source [3]. While diverse compounds were produced in microbes, foreign pathways compete with the endogenous metabolism in the hosts, affecting cell growth and impairing product titer. To achieve higher productivity, various approaches have been used to optimize enzyme expression and carbon flux distribution, including protein engineering to increase the enzyme activity [11], tailoring enzyme expression through ribosome binding site (RBS) or promoter engineering for optimized strength [12,13], and host engineering to block the competing pathways to hijack carbon source for cell production [14–18].

Although these techniques have improved the productivity of microbial cell factories, issues continue to hinder the establishment of efficient microbial systems. One such issue is the lack of real-time regulation in heterologous pathways, leading to unbalanced enzyme expression and metabolic congestion, which impair productivity [19]. Additionally, some heterologous reactions compete with the endogenous essential pathways for precursors or co-factors, resulting in conflicts between cell growth and production [20,21]. However, deleting these competing pathways directly jeopardizes cell growth as well as productivity [22]. Furthermore, the gradual accumulation of toxic intermediates during the biosynthesis of target products in some pathways can cause growth retardation [23]. In such cases, delaying the expression of heterologous genes, downregulating the expression of endogenous essential genes, or minimizing the synthesis of toxic intermediates during the fermentation were required for constructing productive microbial cell factories. Thus, dynamic pathway regulation, including two-phase dynamic regulation and autonomous dynamic regulation, has been developed to effectively solve those problems through designed gene regulation circuits. In the two-phase dynamic regulation, the fermentation is manually split into two phases: a growth phase for biomass accumulation, followed by a production phase for heterologous pathway expression. The shift from growth to production is regulated by adding extracellular inducers, including chemical inducers and physical inducers, at pre-determined times to trigger the dynamic controller for production activation and competing pathway repression. In autonomous dynamic regulation, specific gene activation and repression can be initiated by the cells without manual control. With cell growth, the carbon flux can be autonomously siphoned into heterologous pathways by an intracellular inducer-triggered dynamic controller, mimicking the “just-in-time transcription” prevalent in natural metabolic networks [24]. Additionally, the dynamic controller can autonomously repress the expression of competing genes, enhancing the carbon flux towards target products. Both two-phase dynamic regulation and autonomous dynamic regulation require a dynamic controller, which contains a signal to reflect cellular metabolisms such as pH, temperature, light, and metabolites, a biosensor to detect the signal, and a control valve (promoter) to process the sensor input and transform it into specific output [25].

Here, we reviewed recent advances in dynamic regulation to improve the biosynthesis of value-added compounds. Notable examples in dynamic regulation that increased the productivity of microbial cells are listed in Table 1. We first summarized studies on two-phase dynamic regulation enabled by inducible systems. Next, we analyzed the examples of autonomous dynamic regulation triggered by intracellular signals. Based on the difference in control logic, the common autonomous dynamic regulations were categorized into positive feedback control-based dynamic regulation, oscillation-based dynamic regulation, and the multi-functional dynamic regulation. Finally, future perspectives and outlooks in dynamic regulation were discussed.

## 2. Two-phase Dynamic Regulation: Inducer-triggered Switch from Growth to Production

**Figure 1.**
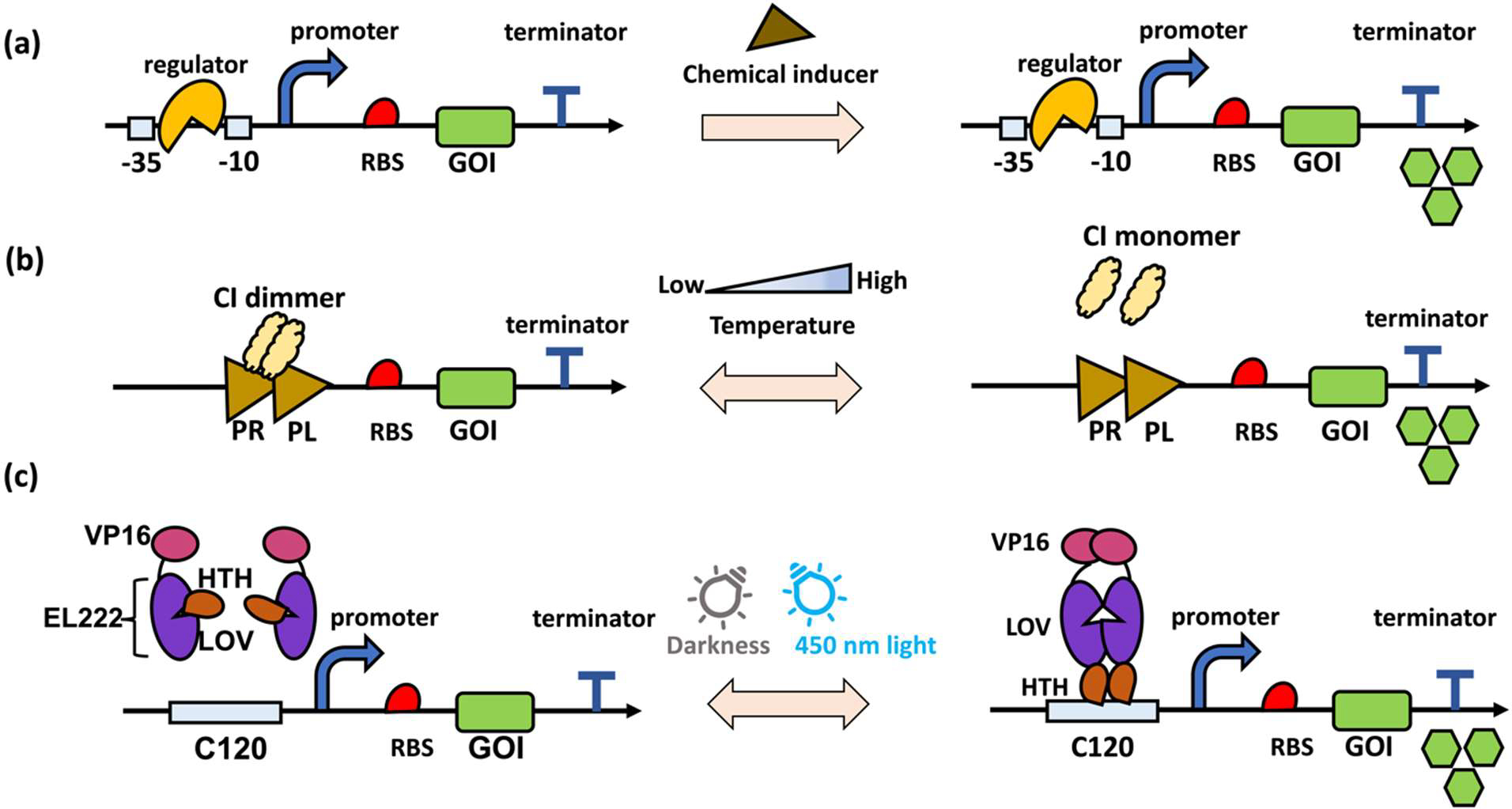
Schemes of two-phase dynamic regulation. **(a)** Chemical inducer-triggered two-phase dynamic regulation. The regulators occupy the −10 and −35 regions and block the access of RNA polymerase. The addition of specific chemical inducers will result in a conformational change of the regulator, making it unable to bind the DNA sequence and thus RNA polymerase will bind and start transcription. **(b)** Temperature-triggered two-phase dynamic regulation. Using the PR/PL-CI system as an example, the promoter PR/PL is repressed by a thermosensitive transcriptional regulator CI dimmer at 30 °C. Increasing the temperature to 37 °C results in a conformational change and the CI monomer will release the repression on promoter PR/PL. **(c)** Light triggered-two phase dynamic regulation. In the darkness, the promoter cannot work normally without the binding of VP16-EL222 complex. The conformational change of VP16-EL222 complex that is triggered by 425 nm blue light can activate the promoter C120. VP16-EL222: a fusion of EL222 with the transcriptional activation domain of VP16 and a nuclear localization signal; HTH: helix-turn-helix DNA-binding domain. LOV: Light-oxygen-voltage.

Decoupling cell growth and production is a predominant strategy to relieve the conflict between the endogenous metabolism and heterologous pathways by delaying the expression of pathway genes or repressing competing pathways until the addition of inducers at a pre-determined time. The commonly used triggers are chemical inducers and physical inducers, as shown in Figure 1.

Two excellent review articles have summarized the induction systems utilized in microbial hosts and their application in dynamic regulation [56,57]. In particular, aTC and IPTG are commonly used inducers to produce valuable compounds in *E. coli*, such as anthocyanin, isopropanol, 1,4-butanediol, and malate [26–28,58] (Figure 1). Leveraging the glucose-repressed and galactose-activated GAL10 and GAL1 promoters increased the productivity of artemisinin and other value-added products in *S. cerevisiae* [32,59]. In addition to the classical induction systems, pH can be used as the chemical inducer for decoupled cell growth and production [60]. For instance, pH-responsive promoters PYGP1 and PGCW14 were employed to regulate lactic acid biosynthesis in *S. cerevisiae* under acidic conditions. The increased accumulation of lactic acid would strengthen the promoter strength and promote a higher titer of lactic acid, resulting in a 10-fold increase compared to the strain with regular promoter [33]. Although chemical inducers effectively split the fermentation process into two stages to buffer the conflict between cell growth and production, the addition of chemical inducers irreversibly changes the cell expression modes. Moreover, the addition of large amounts of chemical inducers is economically unfriendly in industrial applications.

Temperature is a controllable environmental factor that can be rapidly applied and removed from the growth process multiple times [61]. The typical temperature-sensitive promoter, PR/PL, is repressed by a thermosensitive transcriptional regulator CI at 30 °C and activated at 37 °C [62,63] (Figure 1b). In application, the genes for glucose utilization were placed under the control of the PR/PL promoter, which represses their activity during the early stage of fermentation. This allows the *E. coli* cells to concentrate on biomass accumulation. The temperature was then switched to 42 °C when the cell reached the stationary stage to activate the genes related to glucose utilization and ethanol production. Such temperature-triggered positive feedback control circuits increased the ethanol productivity by 3.8 folds compared with the strain without temperature induction [34]. Inspired by this, Fang and colleagues designed a thermal switch using PR/PL to balance the distribution of pyruvate and oxaloacetate between the TCA cycle and L-threonine biosynthesis in *E. coli* [35]. The temperature-trigged positive feedback control was also implemented for itaconic acid [36] and biopolymers polyhydroxyalkanoates biosynthesis in *E. coli* [64]. Although the temperature is easy to control and can work in a plug-and-play manner, exposing the cell to suboptimal temperatures might affect the activity of the endogenous enzyme as well as cell growth. Thus, other environmental inducers are also explored to overcome the limitations of temperature-triggered positive feedback control circuits.

Light inducible circuits have been designed to control gene expression due to the advantage that the pulse and duration time can be precisely controlled without affecting the performance of other endogenous enzymes. Generally, light-inducible circuits leverage light-sensitive proteins that can respond to light and cause corresponding promoters to turn on or off. For example, the EL222 optogenetic transcription system, including light-inducible protein EL222 and its corresponding C120 promoter (P_C120_), was developed to drive the gene expression. In a dark environment, EL222 is unable to bind DNA, whereas exposure to blue light triggers a photochemical reaction between its LOV (Light-oxygen-voltage) domain and flavin chromophore, which activates the HTH domain to combine with DNA and initiate gene transcription. This light-sensitive system was successfully utilized in both T cell and zebrafish [65] (Figure 1c). Such a light-induced circuit was inverted in a manner akin to the NOT logic gate, in which blue light causes gene repression and darkness causes gene activation. The competing gene *pdc* was controlled by a light-inducible system, and the biosynthetic gene *ILV2* was controlled by a dark-inducible system for isobutanol biosynthesis in *S. cerevisiae*. Thus, optogenetic circuits were utilized to drive two phases of cell metabolism: a growth phase with mainly cell growth in blue light, and a production phase in which the cell is devoted to isobutanol production in darkness. The final isobutanol titer showed a 1.6-fold increase compared with the group without light induction [37]. Other light-sensitive factors were also developed as optogenetic circuits and implemented in dynamic regulation. For instance, a red light-controllable system in *S. cerevisiae* was developed to control gene expression, using the light-sensitive dimerization of the PhyB photoreceptor and its interacting partner PIF3 from *Arabidopsis thaliana* [66,67]. The FixJ/FixK2 system was characterized and applied in the mevalonate and isobutanol biosynthetic pathways by light-triggered positive feedback control in *E. coli*. As a result, the final titer showed a 24% and 27% increase, respectively, compared with the group without light control [38]. The CcsA/CcsR system, which originated from *Cyanobacteria*, was optimized to function in *E. coli* to balance the flux distribution between Embden-Meyerhof-Parnas (EMP) and oxidative pentose phosphate (oxPP) pathways [68]. Similarly, the CcsA/CcsR system was also implemented to increase polyhydroxybutyrate (PHB) production in *E. coli* [39]. Nevertheless, the application of light-inducible systems in dynamic regulation has its challenges. The high cell density usually associated with microbial fermentations might limit light penetration, and the effect of light-triggered output will be interfered by constantly changed biomass. In future research, we expect the light-triggered circuit be engineered into a darkness-induced circuit. Cells will prioritize growth in the early stage of fermentation without darkness conditions, and the production phase will be activated by darkness to avoid the problems limiting light penetration in high cell density.

## 3. Autonomous Dynamic Regulation

The aforementioned strategies, though demonstrated successes in enhancing the production in many cases, require external supervision and substantial optimization processes to determine the optimal time and strength for dynamic regulation. However, if cells could autonomously control gene expression in a dynamic manner, the engineered host would self-regulate the pathway flux for growth and production based on its physiological status, maximizing biosynthesis efficiencies, and minimizing the need for human supervision in fermentation processes. Autonomous dynamic pathway regulation, which endows microbes to self-regulate gene expression and cell behavior depending on internal metabolite concentrations or external environment changes, throws light on addressing these problems [69]. The gene of interest can be controlled by dynamic controllers that are able to sense the metabolic state of the cell, coordinating the expression of heterologous genes and endogenous competing genes autonomously [70]. Such a dynamic controller can alleviate the imbalance of foreign gene expression, relieve competition from native essential pathways, and minimize the accumulation of toxic intermediates, resulting in more efficient and effective biosynthesis.

### 3.1. Positive Feedback Control-based Autonomous Dynamic Regulation

**Figure 2.**
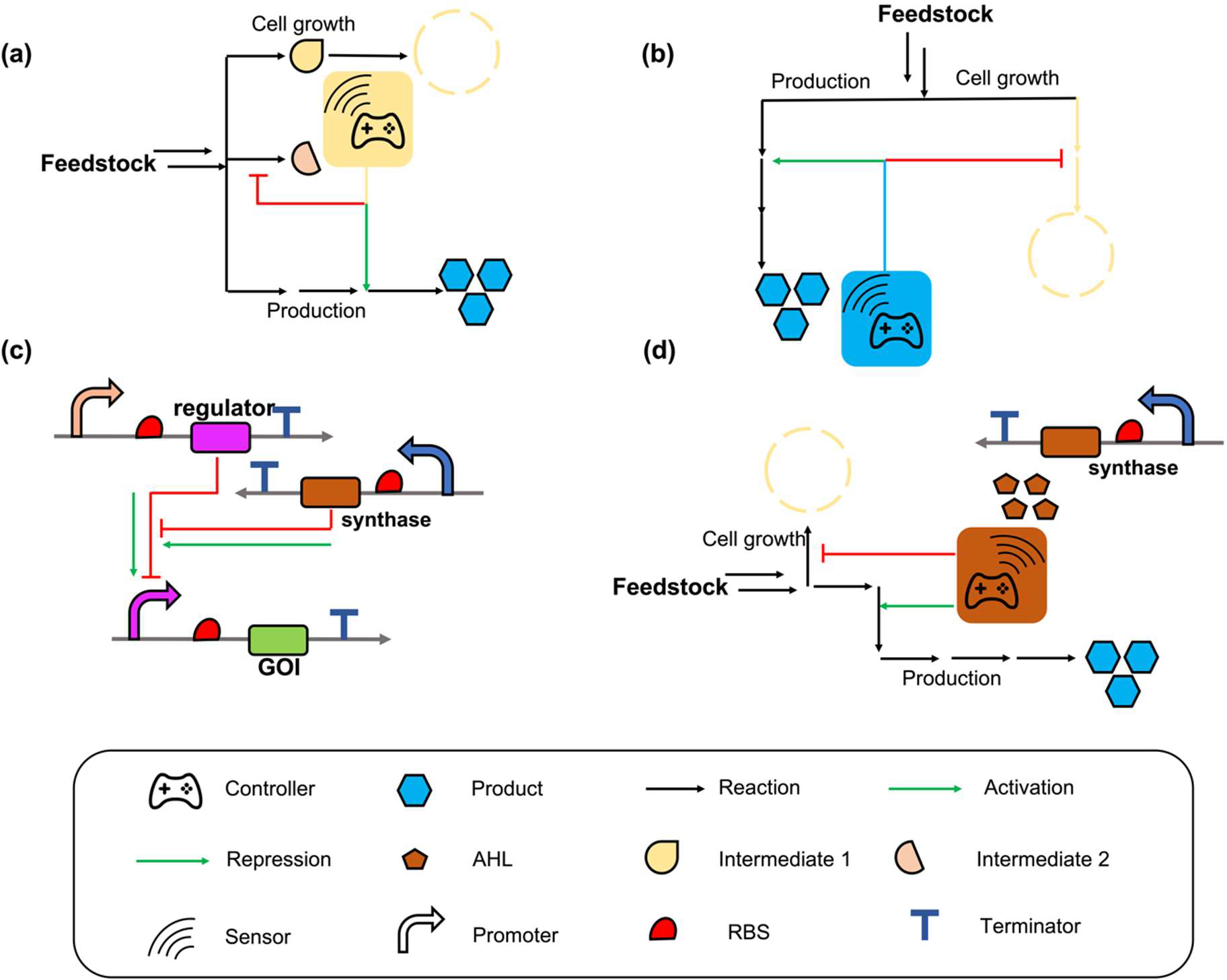
Schemes of positive feedback control-based autonomous dynamic regulation. **(a)** Cellular metabolite-based positive feedback control. With cell growth, the increased concentration of the specific metabolite will strengthen the output of the control valve, together with strengthening related gene activation or repression to promote production, forming a positive feedback control-based dynamic regulation. **(b)** Final product-triggered feedback control. The genes that are responsible for product biosynthesis and genes that compete with heterologous pathways are under the control of the product-triggered promoter. With the accumulation of the final product, the related genes will be activated or repressed autonomously. **(c)** The mechanism of QS system. The AHL (3-oxohexanoylhomoserine lactone) synthesized by AHL synthase can release the repression by a related repressor, and the promoter can work normally. **(d)** QS system-triggered positive feedback control. Key genes are under the control of a QS-triggered promoter. With the accumulation of AHL, the genes can be activated or repressed autonomously.

Positive feedback control is a process occurring in a feedback loop, amplifying the effect of output on the system. Implementing the positive feedback control logic for autonomous dynamic regulation to control genes can magnify the activation or repression level with the accumulation of inducers during cell cultivation. With the accumulation of specific inducers, the genes responsible for the production and competing pathways that impair productivity will be dynamically and persistently upregulated and downregulated, respectively. Positive feedback control-based autonomous dynamic regulation can typically be triggered by specific metabolites or quorum sensing (QS) systems (Figure 2).

Metabolites are straightforward indicators that reflect the cellular metabolism and are typically applied as inducers to control gene expression in autonomous dynamic regulation. Metabolite-based biosensors have been developed to up- and down-regulate genes related to biosynthesis. With cell growth, the enhanced concentration of a specific metabolite increases the output of the control valve, together with strengthened gene activation or repression to enhance the production of target compounds, forming a positive feedback control-based dynamic regulation. Farmer and colleagues first reported the concept of positive feedback control-based pathway dynamic regulation in 2000. They developed a dynamic controller that senses the endogenous compound acetyl phosphate for lycopene biosynthesis in *E. coli* [25]. In another study, Wu and colleagues designed an autonomous dual-control system based on the intermediate glucosamine-6-phosphate biosensor, enabling cells to self-adjust carbon flux for the synthesis of high-value nutraceutical N-acetylglucosamine in *Bacillus subtilis*. Growth-related competing genes *pfkA*, *zwf*, and *glmM* were autonomously repressed, and the gene GAN1, responsible for N-acetylglucosamine biosynthesis, was autonomously activated with the accumulation of glucosamine-6-phosphate, showing a 1.6-fold increase in the final N-acetylglucosamine titer in a 15 L fed-batch bioreactor [40]. Similarly, a genetic switch that can sense the cell circle changes from the exponential growth phase to the stationary phase was designed to toggle the gene expression pattern for γ-aminobutyric acid (GABA) biosynthesis in *C. glutamicum*, representing a 58.9% increase in the final GABA titer [41].

In addition to the above, final products can serve as signals indicating that cells are in production mode, and dynamic regulation can be executed to coordinate gene expression. For example, *p*-coumaric acid is a valuable aromatic compound that serves as a significant precursor for the synthesis of naringenin, resveratrol, and apigenin [71,72]. Despite the high value of *p*-coumaric acid, the metabolic pathway of *p*-coumaric acid competes with cell growth for PEP (phosphoenolpyruvate) and E4P (erythrose 4-Phosphate). To solve this problem, Li et al. developed *p*-coumaric acid-triggered positive feedback control circuits powered by the PadR biosensor system to fine-tune the metabolism in *E. coli*. The plasmid, which contained enzymes (TAL, TyrA*, PpsA, TktA, and AroG*) to strengthen *p*-coumaric acid production and asRNA which represses competing gene *ppc* to drive more PEP toward the shikimate pathway for *p*-coumaric acid biosynthesis, was designed first [43]. By using a *p*-coumaric acid-triggered promoter to control the replication of the plasmid, the copy number of the plasmid would be kept at a normal level when no *p*-coumaric accumulates [73]. With the gradual accumulation of *p*-coumaric acid, the plasmid was activated to replicate at a high level so that the pathway genes were further overexpressed and competing genes were repressed. The *p*-coumaric acid trigged feedback control circuits increased the *p*-coumaric acid tier by 77.89% compared to the strain with static regulation [42]. In this case, the final product-triggered positive feedback control was designed not only to balance the heterologous enzyme expression but also to relieve the competition between cell growth and production. The final product-triggered positive feedback control was also used in the synthesis of muconic acid [43], vanillin [44], and other value-added products in *E. coli* or *S. cerevisiae* [45,46,74]. While the metabolite-trigged positive feedback control circuit can manipulate cell metabolism, especially the balance of heterologous genes and competing genes, there are still some limitations that restrict its application in dynamic regulation. One major limitation of metabolite-based positive feedback control is finding appropriate biosensors that could respond to the desired metabolite. To overcome this limitation, firstly, advances in transcriptional and translational omics can provide new knowledge for mining new biosensors [75]. Analyzing the natural regulation networks in various organisms can inspire researchers to characterize new transcriptional factor-based biosensors. Secondly, the development of bioinformatic tools such as the machine learning-based structure prediction program AlphaFold [76] provides effective approaches to obtain the structure of existing biosensors. At the same time, computational technologies such as molecular dynamic simulation and molecular docking allow *in silico* analysis of the interaction between biosensors and inducers, which provides a theoretical prediction to engineer existing biosensors for increased substrate scope [77]. Overall, increasing the number of available biosensors is paramount for overcoming the current limitations of metabolite-triggered feedback control-based dynamic regulation.

Quorum sensing (QS) system functions to manipulate gene expression in accordance with the cell density [78,79]. A typical QS system is composed of three components: (1) an enzyme responsible for the synthesis of inducer as the cell grows, (2) a regulator that respond to the inducer, and (3) a promoter that can be regulated by the regulator for gene activation or repression [80,81]. In *E. coli*, the reported QS systems used in positive feedback control-based dynamic regulation include LuxR/LuxI system from *Vibrio fscheri* [82], EsaR/EsaI system from *Pantoea stewartia* [83], and SrbR/SrbA system from *Streptomyces rapamycinicus* [84]. Prather and colleagues have successfully developed the LuxR/LuxI and EsaR/EsaI systems and implemented them to produce valuable compounds including glucaric acid, *myo*-inositol [85], naringenin, and salicylic acid [48]. For example, they engineered the EsaR/EsaI system in *E. coli* to create circuits that can turn off gene expression at the desired time and cell density with the desired strength. By integrating these circuits into *E. coli*, they enabled positive feedback control of endogenous essential genes (*pfk-1* and *aroK*) in glycolysis and aromatic amino acid biosynthesis, which compete with the biosynthesis of myo-inositol and shikimic acid [31,86]. The final titer of *myo*-inositol showed a 5.5-fold increase, and the shikimic acid titer increased from unmeasurable to 100 mg/L compared with the group without dynamic regulation [47]. In 2022, Ge and colleagues engineered the LuxR/LuxI system and created a QS library that showed versatile dynamic performance in different cell densities. The engineered LuxR/LuxI circuits were utilized to dynamically downregulate the competing gene *ppc* and upregulate the salicylic acid biosynthetic genes in *E. coli* [87], causing a 2.1-fold increase in salicylic acid titer compared to the static control. These engineered circuits were also used to coordinate gene expression in the biosynthesis of 4-hydroxycoumarin, which is a valuable compound for the production of anticoagulant drugs [88,89], resulting in an 11.3-fold increase compared to the static control [49].

In addition to the application in *E. coli*, QS systems have also been developed in *S. cerevisiae* and *B*. subtilis for controlling gene expression based on cell density [79,90]. For example, a typical QS system was designed by combining the endogenous yeast Ypd1-Skn7 signal transduction pathway with a plant hormone cytokinin system. In the cytokinin-based QS system, isopentenyladenine (IP), which was produced through the isopentenylation of ATP by AtIPT4 from *A. thaliana*, acted as a growth-related indicator that triggered the phosphorylation of Ypd1-Skn7 system, activating the SSRE promoter for gene expression. At high population density, the QS system repressed the competing genes for FPP consumption and more FPP would be siphoned for α-farnesene biosynthesis, resulting in an 80% increase in the final titer [50]. Although QS systems have been well characterized in dynamic regulation for increasing the titer of the products of interest and can be utilized in the pathways without specific biosensors, the introduction of heterologous genes, particularly those for inducer biosynthesis, hijacked a part of cell resource. Furthermore, QS system reflects the behavior of the entire group of cells rather than the cellular status in individual cells. Changes in cultivation conditions can result in different growth curve and deferent regulation pattern, which can affect the regulation efficiency of the QS system. These characteristics limit the further applications of QS system in dynamic regulation. Both metabolite- and QS-based positive feedback control enable cells to autonomously switch from growth to production mode.

### 3.2. Oscillation-based Autonomous Dynamic Regulation

**Figure 3.**
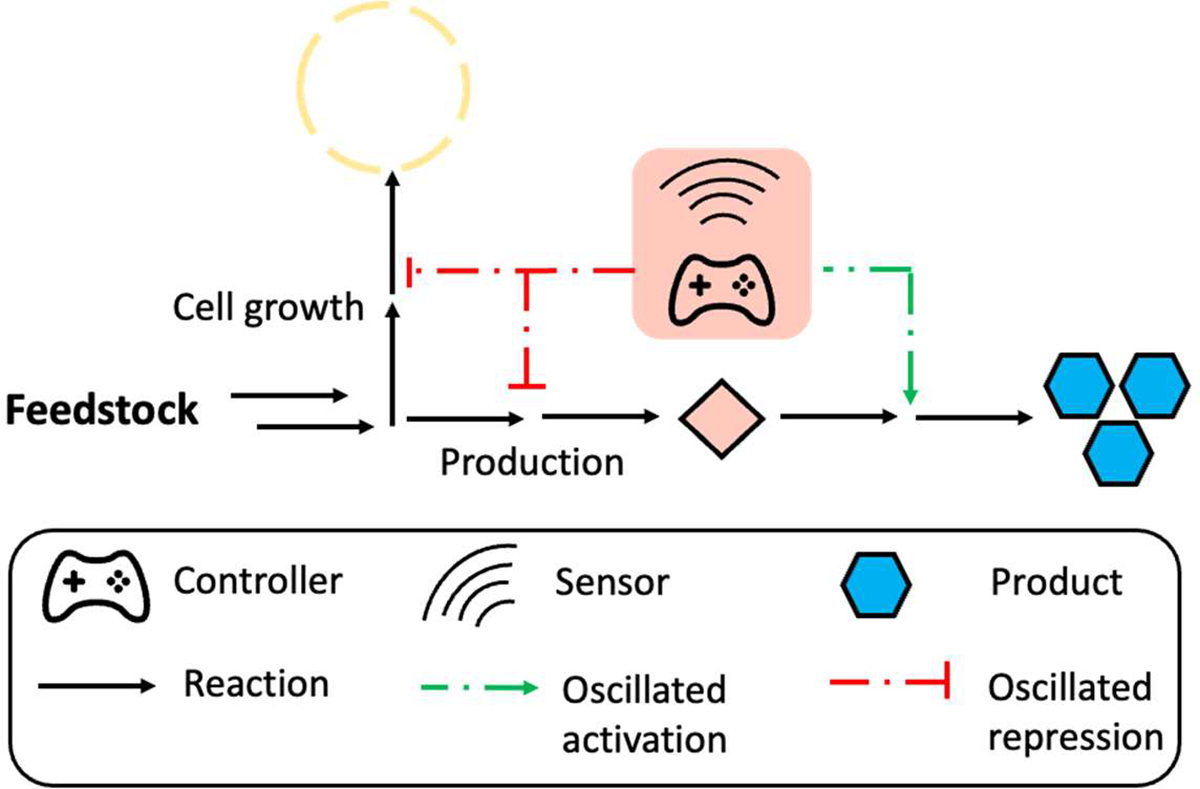
Schemes of oscillation-based autonomous dynamic regulation. The gradual accumulation of a specific intermediate will trigger the downstream consumption pathway for production as well as repression of the upstream formation pathway and competing pathway. With the intermediate consumption for production, decreased concentration of intermediate will turn off downstream consuming genes, and at the same time turn on upstream genes for intermediate formation, forming an oscillated gene activation and repression.

While positive feedback control is a one-way regulation where the output signal is continuously amplified once the regulation starts, oscillation-based autonomous dynamic regulation is usually triggered by an intermediate in biosynthetic pathway and consists of both up- and down-regulation on one gene. Unlike the final product, the intermediate concentration changes with its relative production and consumption rate, resulting in an oscillated output of the related promoter. This regulation logic allows the host cells to autonomously coordinate gene activation or repression in response to ever-changing environments (Figure 3). In practice, pyruvate is a key intermediate that links glycolysis to the tricarboxylic acid (TCA) cycle and is also the precursor for glucaric acid biosynthesis in *B. subtilis*. Knocking out competing genes for TCA cycle impaired cell growth as well as glucaric acid production. Xu and colleagues developed a pyruvate biosensor-based oscillation circuit to regulate the glucaric acid pathway in *B. subtilis*. The increased pyruvate accumulation triggered the repression for pyruvate formation and increase its consumption for biosynthesis of the glucaric acid. Then, with the decrease of pyruvate concentration, the repression of pyruvate generation and enhancement on pyruvate consumption to glucaric acid would be inhibited, causing an oscillated concentration of pyruvate. This oscillation-based dynamic pathway regulation led to a 2.5-fold increase in glucaric acid titer compared with the static control [51]. In another example, Xu et al designed an oscillation-based autonomous dynamic regulation circuit for fatty acid biosynthesis. The accumulation of malonyl-CoA could activate the gene *ACS*, converting malonyl-CoA to fatty acids, while at the same time, the *ACC* gene which is responsible for malonyl-CoA formation was repressed. With the consumption of malonyl-CoA, the repression of the malonyl-CoA biosynthesis pathway was relieved, and the consumption pathway would be repressed again. The intracellular malonyl-CoA showed an oscillatory changing pattern in the strains with malonyl-CoA controller, and the final fatty acid titer exhibited a 2.1-fold increase compared with the strain without dynamic control in *E. coli* [52]. Inspired by these findings, Liu and colleagues designed an oscillation circuit based on *p*-coumaroyl-CoA and malonyl-CoA biosensors for naringenin biosynthesis in *E. coli*, resulting in a 15-fold increase over the nonregulated system in naringenin titer [91].

Oscillation-based dynamic regulation can not only autonomously balance heterologous enzyme expression and repress competing pathway expression, but also reduce the growth retardation caused by toxic intermediates in some pathways [23]. Overproduction of toxic intermediates leads to impaired cell growth, reduced yield and productivity [92]. An ideal regulation system for such pathways can sense the cell status and dynamically control the consumption and production of toxic intermediates to reduce their excessive accumulation and relieve growth retardation. To achieve this, an intermediate-based biosensor is required to control the genes for toxic intermediate formation and consumption. During the early stage of fermentation, most of the cell resources are used for growth, and the genes for production are repressed, leading to the gradual accumulation of toxic intermediate. Once the biosensor threshold is achieved, the genes for toxic intermediate consumption, along with the genes for product formation are activated, and the genes responsible for toxic intermediates formation are repressed to avoid the excess accumulation that impairs cell growth. This metabolic mode decreases the intermediate concentration so that the genes for the consumption will be turned off and genes for the formation will be turned on to accumulate the intermediates again, forming an oscillated dynamic regulation [93]. The oscillation-based dynamic regulation system enables cells to coordinate intermediate consumption and formation autonomously. In 2013, Dahl and colleagues designed an FPP-responsive oscillation circuit in *E. coli* to produce amorphadiene, the precursor of artemisinin that is well-established for the treatment of malaria. The intermediate (FPP) for artemisinin biosynthesis is toxic when it accumulates in *E. coli*. In this control logic, the gene *ADS* for artemisinin biosynthesis was controlled by an FPP-responsive promoter, and the accumulation of FPP triggered the expression of *ADS* to consume FPP, as well as repress the upstream genes for FPP formation to keep FPP at a low level and decrease the toxicity. The final artemisinin titer showed a 2-fold increase compared to the group without dynamic regulation [23]. Similarly, Shen and colleagues used FPP as a trigger to strengthen its consumption and decrease its formation in zeaxanthin biosynthetic pathway, resulting in a 2-fold increase in the final titer compared with the static control in *E. coli* [53].

While the oscillation-based autonomous dynamic regulation enables cells to intelligently control gene expression and minimize the toxicity of intermediates accumulation, it has some limitations. Similar to metabolite-based positive feedback control, oscillation-based dynamic regulation requires specific inducers in response to intermediates. The limited number of biosensors and narrow substrate scope of existing biosensors restricts the application of oscillation-based dynamic regulation. Moreover, the oscillated mode in cell metabolism may cause unstable cell performance along with decreased robustness.

### 3.3. Multi-functional Autonomous Dynamic Regulation

**Figure 4.**
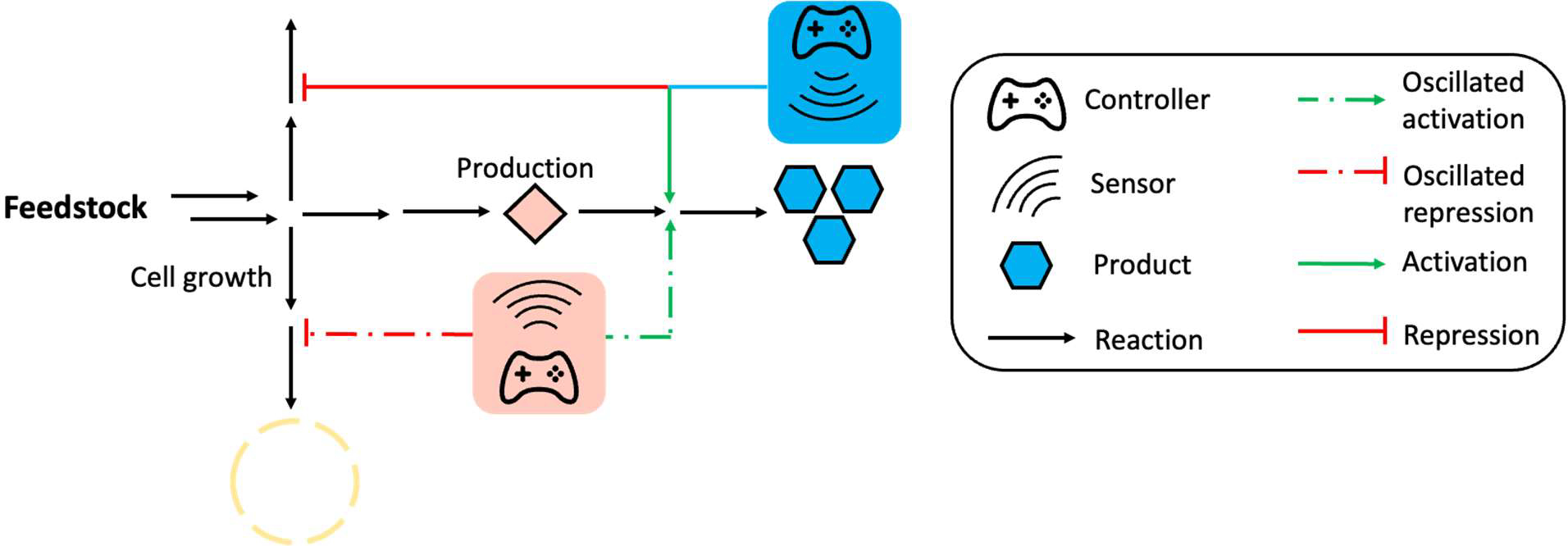
Schemes of multi-functional autonomous dynamic regulation. More than one biosensor and control logic were used to coordinate gene expression and repression. As shown in the figure, this multi-functional autonomous dynamic regulation network includes intermediate-triggered oscillation and final product-triggered positive feedback control.

Although single logic dynamic controls, including positive feedback control and oscillation-based dynamic regulation, have been proven effective in balancing heterologous enzyme expression, minimizing competition between cell growth and production, and reducing the accumulation of toxic intermediates, such regulation can become ineffective when facing complicated pathways that require complex metabolic control. Therefore, combining multiple biosensors for complex dynamic regulation is needed to address the challenges in complicated pathways. This section focuses on discussing several representative cases where multiple biosensors and dynamic regulation strategies were combined to establish a sophisticated dynamic control network for high titers of target compounds (Figure 4). For example, the positive feedback control and feedforward control circuits were combined to coordinate the genes expression in naringenin biosynthesis pathway. Naringenin is a plant-source flavonoid with antibacterial, antiviral, and antifungal prosperities, and has been explored in the treatment of Alzheimer’s disease [94–96]. The biosynthesis of naringenin in *E. coli* requires four enzymes: tyrosine ammonia lyase (TAL), 4-coumaryl-CoA ligase (4CL), chalcone synthase (CHS), and chalcone isomerase (CHI) [97]. Due to unbalanced enzyme expression and competition for malonyl-CoA with fatty acid biosynthesis, the productivity of naringenin biosynthesis pathway was limited in *E. coli* [98–100]. Researchers designed an Autonomous Cascaded Artificial Dynamic (AutoCAD) regulation system to mimic the natural regulation in cells and fine-tune the heterologous enzyme expression in naringenin pathway. In the AutoCAD regulation system, *p*-coumaric acid, one of the essential intermediates, was used as the first inducer to trigger the expression of rate-limiting enzymes (4CL and CHS), which formed the feedforward regulation circuit, resulting in a 10.4-fold (from 12.1 mg/L to 125.8 mg/L) increase in naringenin titer. Next, naringenin was used as the second inducer to further increase the expression of the key enzyme (CHS) forming the positive feedback regulation-based dynamic regulation. The accumulation of naringenin strengthened the expression of CHS, enhancing the naringenin titer to 148.3 mg/L. Finally, the *p*-coumaric acid was further used as inducer to repress the competitive consumption of malonyl-CoA by the fatty acid biosynthesis pathway and rewired more malonyl-CoA for naringenin biosynthesis, forming an additional layer of positive feedback control. By combining all the control logics together, the final naringenin titer showed a 16.5-fold increase. The concept of AutoCAD regulation system that contains both intermediate-based feedforward control and product-triggered feedback control was successfully designed to coordinate heterologous enzyme expression and rewire more malonyl-CoA for naringenin biosynthesis [54]. Zhou et al. also designed the *p*-coumaric acid-based oscillation circuits and naringenin-triggered positive feedback control circuits to rewire more malonyl-CoA for naringenin biosynthesis in *E. coli*, which showed an 8.7-fold increase in naringenin titer with the addition of tyrosine, a direct precursor of naringenin biosynthesis [55]. Similar strategy was applied in yeast to improve the strain stability and naringenin pathway yield. A malonyl-CoA-based biosensor was developed to dynamically decrease the consumption of malonyl-CoA for fatty acid. The increased accumulation of malonyl-CoA would strengthen the repression of genes for fatty acid biosynthesis and rewire more malonyl-CoA for naringenin pathway, which formed a feedforward control circuit. At the same time, a naringenin-based biosensor was utilized to control the leucine synthesis gene, which is essential for cell fitness. Only cells that produce naringenin can activate the biosynthesis of leucine synthase to support cell growth. Such a positive feedback control circuit decreased the amounts of lazy strains during the fermentation, thereby increasing the strain stability [101]. Combining multiple biosensors and regulation logics can overcome the limitations of single biosensor-based dynamic regulation and has the potential to be applied in engineering the complicated pathways in microbial cell factories. However, limitations exist when combining multiple biosensors and regulation logics in one cell. Similar to metabolite-based single biosensor dynamic regulation, the limitations of biosensors can restrict the development of multiple biosensor-based dynamic regulation. Additionally, the combination of more than one control logics in one cell can cause instability, and the application of multiple biosensors may cause cross-talk effects, which can affect the related output as well as the regulation effect.

## 4. Concluding Remarks and Furfure Perspectives

Dynamic regulation is a promising approach for coordinating heterologous gene expression and rearranging the expression of endogenous competing genes to increase the productivity of microbial cell factories. By mimicking the natural metabolic network, the dynamic regulation circuits were applied in the engineered host, endowing cells the intelligence to coordinate gene expression and repression autonomously. As summarized in this review, two-phase dynamic regulation and autonomous dynamic regulation, including positive feedback control, oscillation, and multi-functional dynamic control, have been applied ubiquitously in metabolic engineering to achieve remarkable improvements in titers and yields of value-added products [57].

Each regulation logic has its advantages and disadvantages, which have been pointed out in related sections. One obvious factor that constrains the application of dynamic regulation is the limited number of biosensor systems available as controllers. As discussed in the sections of positive feedback control-based and oscillation-based dynamic regulation, new biosensors can be characterized or engineered to sense an increasing number of metabolites to expand the applicable scenarios of dynamic regulation. Besides mining for novel biosensor systems, another way to address the limited number of biosensors is to mine and develop central metabolites-based biosensors (for example, acetyl-CoA and pyruvate) [51]. As most of the microbial biosynthetic pathways start from central metabolites, developing central metabolite-responsive biosensor systems would further expand the applicable scenarios of dynamic regulations, although there are still challenges on utilizing these central metabolites-based biosensor. First, such central metabolites-triggered dynamic controller may start the autonomous regulation early due to the earlier accumulation of inducers. Moreover, the concentrations of central metabolites are often tightly regulated by endogenous regulation networks, and the integration of heterologous pathways will result in diverse interference with the accumulation of central metabolites, causing fluctuated dynamic regulation performance.

Furthermore, endogenous natural pathways are regulated at DNA, RNA, and protein level to provide reasonable and timely control. However, most of the dynamic regulation exerted in metabolic engineering is at the DNA or RNA level [57,102]. For gene activation, the dynamic control at DNA or RNA level will be more straightforward and effective in starting gene expression, but gene repression will be less effective at the DNA or RNA level, especially when the translated proteins are stably maintained in the cells [16]. These proteins can still work normally, which affects the regulation effect. Developing an expansive set of protein level dynamic regulation tools, such as protein degradation tags, degrons, or proteases, achieves fast and accurate control toward target proteins, which may boost the efficiency of dynamic regulation and optimization of engineered metabolic pathways [58,103,104]. In the future, dynamic regulation will see combinatory optimization by combing diverse control logics at DNA, RNA, and protein level.

## Figures and Tables

**Table 1.** Summary of notable examples in dynamic regulation to increase the productivity of microbial cells.

Inducer	Control Logic	T arget	Product	Organism	Achievement	Ref.
**IPTG/aTc**	Two-phase	*PC, CS, ACN, ICL, MS*	malate	*E. coli*	2.3-fold (titer)	[26]
**IPTG**	Two-phase	*metJ*	peonidin 3-O-glucoside	*E. coli*	21-fold (titer)	[27]
**IPTG**	Two-phase	*gltA*	isoprenol	*E. coli*	3.7-fold (titer)	[28]
**aTc**	Two-phase	*gabD, ybgC, tesB*	1,4-BDO	*E. coli*	~2-fold (titer)	[29]
**aTc**	Two-phase	*Pfk*	glucaric acid	*E. coli*	42% (titer)	[30]
**aTc**	Two-phase	*Pfk*	myo-inostiol	*E. coli*	2-fold (titer and yield)	[31]
**Glucose/galactose**	Two-phase	*ERG9, tHMG1, CrtE, CrtYB, CrtI*	carotenoids	*S. cerevisiae*	1156 mg/L (titer)	[32]
**pH**	Two-phase	*idhL*	lactic acid	*S. cerevisiae*	2.7-fold (titer)	[33]
**Temperature**	Two-phase	*ptsG*	ethanol	*E. coli*	3.8-fold (titer)	[34]
**Temperature**	Two-phase	*IdhA, pflB, poxB, ptA, adhE*	L-threonine	*E. coli*	1.4-fold (yield)	[35]
**Temperature**	Two-phase	*ICD*	itaconic acid	*E. coli*	22% (productivity)	[36]
**Light**	Two-phase	*pdc*	isobutanol	*S. cerevisiae*	1.6-fold (titer)	[37]
**Light**	Two-phase	*AtoB, HMGS, tHMGR*	mevalonate	*E. coli*	23% (titer)	[38]
**Light**	Two-phase	*gltA, phbABC*	polyhydroxybutyrate	*E. coli*	3-fold (titer)	[39]
**Acetyl phosphate**	Positive feedback control	*PPS, Idi*	lycopene	*E. coli*	3-fold (productivity)	[25]
**Glucosamine-6-phosphate**	Positive feedback control	*pfkA, zwf glmM, GAN1*	N-acetylglucosamine	*B. subtilis*	1.6-fold (titer)	[40]
**Cell cycle change**	Positive feedback control	*GAD, GadC*	*γ*-aminobutyric acid (GABA)	*C. glutamicum*	58.9% (titer)	[41]
***p*-Coumaric acid**	Positive feedback control	The plasmid containing TAL, TyrA *, PpsA, TktA, and AroG*	*p*-coumaric acid	*E. coli*	77.89% (titer)	[42]
**Muconic acid**	Positive feedback control	*ppc, pykf, EntC, PchB*	muconic acid	*E. coli*	1.8 g/L (titer)	[43]
**Vanillin**	Positive feedback control	*Fcs, Ech*	vanillin	*E. coli*	~2-fold (titer)	[44]
**Malonyl-CoA**	Positive feedback control	*MCR*_*ca*_	3-hydroxypropionic acid	*S. cerevisiae*	10-fold (titer)	[45]
**L-threonine**	Positive feedback control	*rhtABC*	L-threonine	*E. coli*	161.01%	[46]
**QS system (EsaR/EsaI)**	Positive feedback control	*aroK*	shikimate	*E. coli*	Unmeasurable to 100 mg/L	[47]
**QS system (EsaR/EsaI)**	Positive feedback control	*Pfk-1*	*myo*-inositol	*E. coli*	5.5-fold	[47]
**QS system (LuxR/LuxI, EsaR/EsaI)**	Positive feedback control	*Ics, Ipl*	salicylic acid	*E. coli*	1.8-fold (titer)	[48]
**QS system (LuxR/LuxI, EsaR/EsaI)**	Positive feedback control	*TAL, 4CL*	naringenin	*E. coli*	6.5-fold (titer)	[48]
**QS system (LuxR/LuxI)**	Positive feedback control	*ppc, entC, pchB*	salicylic acid	*E. coli*	2.1-fold	[49]
**QS system (LuxR/LuxI)**	Positive feedback control	*entC, pchB, pqsD, sdgA*	4-hydroxycoumarin	*E. coli*	11.3-fold	[49]
**QS system (Ypd1-Skn7)**	Positive feedback control	*Erg9*	*α*-farnesene	*S. cerevisiae*	80% (titer)	[50]
**Pyruvate**	Oscillation	*zwf, pgi, ino1*	glucaric acid	*B. subtilis*	2.5-fold (titer)	[51]
**Malonyl-CoA**	Oscillation	*ACC, FAS*	malonyl-CoA	*E. coli*	2.1-fold (titer)	[52]
**FPP**	Oscillation	*ADS, atoB, HMGs, tHMGR, MK, PMK, PMD, idi, ispA*	amorphadiene	*E. coli*	2-fold (titer)	[23]
**FPP**	Oscillation	MEV pathway	zeaxanthin	*E. coli*	2-fold (titer)	[53]
***p*-Coumaric acid and naringenin**	Multi-functional dynamic control	*4CL, CHS, fabD*	naringenin	*E. coli*	16.5-fold (titer)	[54]
***p*-Coumaric acid and naringenin**	Multi-functional dynamic control	*ACC, gltA, acs, acpS, acpP, acpT, apcH, fabD,*	naringenin	*E. coli*	8.7-fold (titer)	[55]
